# Intraoperative aberrometry compared to preoperative Barrett True-K formula for intraocular lens power selection in eyes with prior refractive surgery

**DOI:** 10.1038/s41598-022-11462-8

**Published:** 2022-05-05

**Authors:** Suzie A. Gasparian, Saman Nassiri, Hyelin You, Abby Vercio, Frank S. Hwang

**Affiliations:** Loma Linda University Eye Institute, 11370 Anderson Street, Suite 1800, Loma Linda, CA 92354 USA

**Keywords:** Refractive errors, Corneal diseases, Eye diseases, Lens diseases, Medical research, Outcomes research

## Abstract

To compare the predictive refractive accuracy of intraoperative aberrometry (ORA) to the preoperative Barrett True-K formula in the calculation of intraocular lens (IOL) power in eyes with prior refractive surgery undergoing cataract surgery at the Loma Linda University Eye Institute, Loma Linda, California, USA. We conducted a retrospective chart review of patients with a history of post-myopic or hyperopic LASIK/PRK who underwent uncomplicated cataract surgery between October 2016 and March 2020. Pre-operative measurements were performed utilizing the Barrett True-K formula. Intraoperative aberrometry (ORA) was used for aphakic refraction and IOL power calculation during surgery. Predictive refractive accuracy of the two methods was compared based on the difference between achieved and intended target spherical equivalent. A total of 97 eyes (69 patients) were included in the study. Of these, 81 eyes (83.5%) had previous myopic LASIK/PRK and 16 eyes (16.5%) had previous hyperopic LASIK/PRK. Median (MedAE)/mean (MAE) absolute prediction errors for preoperative as compared to intraoperative methods were 0.49 D/0.58 D compared to 0.42 D/0.51 D, respectively (*P* = 0.001/0.002). Over all, ORA led to a statistically significant lower median and mean absolute error compared to the Barrett True-K formula in post-refractive eyes. Percentage of eyes within ± 1.00 D of intended target refraction as predicted by the preoperative versus the intraoperative method was 82.3% and 89.6%, respectively (*P* = 0.04). Although ORA led to a statistically significant lower median absolute error compared to the Barrett True-K formula, the two methods are clinically comparable in predictive refractive accuracy in patients with prior refractive surgery.

## Introduction

Refractive surgery is one of the most common ophthalmic procedures performed in the world^1^. There is significant growth in the number of post-refractive patients who desire cataract surgery given the aging population^2^. Despite excellent outcomes following refractive laser surgery, intraocular lens (IOL) power calculation and visual outcomes following cataract surgery are less predictable in eyes with prior refractive procedures^3,4^. Conventional methods of IOL power estimation do not always perform well in post-refractive patients. It has become increasingly challenging to select the proper IOL power to achieve more accurate post-operative refractive outcomes for patients who expect spectacle independence^5^. In recent years, there has been a growing interest with significant advances in reliable methods of IOL power estimation in this patient population. Some of these methods rely on historical data whereas others require only current measurements, all with varying degrees of success.

One of the more recently incorporated formulas, which does not rely on pre-refractive surgery historical data is the Barrett True-K formula provided by the Asia Pacific Association of Cataract & Refractive Surgeons (APACRS)^6^. The Barrett True-K formula is based on the Barrett Universal II Formula and calculates a modified keratometry value for patients who have had previous myopic or hyperopic LASIK or PRK. It is also able to accurately calculate corneal height when central keratometry has been altered, which has made this formula increasingly reliable.

In addition to preoperative formulas used to calculate IOL power, many surgeons have introduced an intraoperative wavefront aberrometry device (IA; the Optiwave Refractive Analysis [ORA] System; Alcon Laboratories, Inc., Fort Worth, TX, USA) in the operating room, which has led to a real-time aphakic IOL power calculation during cataract surgery. This device shows promise in enhancement of operative outcomes for patients with a history of corneal refractive surgery. This technique does not rely on the corneal power, and thus, it is not prone to errors imposed by previous corneal surgeries or irregularities along with issues related to optical limitations such as a dense cataract^7^. It has shown progressively promising results in visual outcomes through optimal IOL selection and implantation during cataract surgery, especially in challenging post-refractive cases^8^.

The purpose of our study is to compare the predictive refractive accuracy of intraoperative aberrometry (the ORA System) with the preoperative Barrett True-K formula for IOL power calculation in patients with a history of myopic or hyperopic refractive surgery. To our knowledge, there is no data available in the literature comparing these methods in patients with a history of refractive surgery.

## Methods

### Study subjects

This retrospective longitudinal study was performed at the Loma Linda University Eye Institute, Loma Linda, California, USA. This study (#5180084) was exempt by the Institutional Review Board (IRB) at Loma Linda University and was conducted in accordance with the Declaration of Helsinki and Health Insurance Portability and Accountability Act. We reviewed the electronic medical records of patients with a history of myopic or hyperopic laser in situ keratomileusis (LASIK) or photorefractive keratectomy (PRK) who underwent cataract surgery with IOL implantation from October 2016 to March 2020. This study included both eyes (22 total patients—4 hyperopic LASIK, 18 myopic LASIK), when available, of all patients who underwent uncomplicated standard phacoemulsification cataract surgery with IOL implantation in the capsular bag. Patients with the following criteria were excluded from the study: poor visual potential, ocular disease limiting corrected distance visual acuity to worse than 20/40, insufficient follow-up, lack of preoperative, intraoperative, or postoperative data, postoperative refraction less than 1 month after surgery, complicated cataract surgery (e.g. posterior capsule tear/vitreous loss), and uncontrolled intraocular pressure.

### Procedure

Preoperative assessment was completed using optical biometric data derived from IOL Master (IOL Master 500 or 700, Carl Zeiss Meditec, Jena, Germany) as part of routine cataract evaluation. The parameters measured by the IOL Master 700 had a green checklist, which ensured that they were correctly measured. Biometric data was utilized to calculate IOL power and predict post-operative spherical equivalent (SE) through the online Barrett True-K formula provided by APACRS (preoperative method). Variables used to perform IOL power calculation included type of previous refractive surgery (myopic or hyperopic), lens factor or A-constant, axial length, keratometry, anterior chamber depth (ACD), target refraction, including optional variables corneal diameter (WTW) and lens thickness when available. Previously optimized lens constant (Lens factor/A-constant) auto-filled upon selecting the IOL on APACRS Barrett True-K formula website was utilized for all surgeons. Standard phacoemulsification was performed for all patients. Intraoperatively, following removal of lens material, normal intraocular pressure was established by Barraquer (Ocular) tonometer through the injection of a cohesive viscoelastic agent, ProVisc (Alcon, Ft Worth, TX, USA). Intraoperative wavefront aberrometry by the ORA System was used for aphakic intraoperative refraction and IOL power calculation (intraoperative method). The IA provided a recommended IOL power with predicted postoperative SE based on target refraction, and predicted postoperative SE for the actual IOL implanted, if it varied from the recommended IOL power. Results were compared and final spherical power of the IOL was chosen based on the least possible postoperative residual refractive error with a trend toward slight myopia, based on the surgeon’s preference. Intraocular lenses implanted for the patients were monofocal IOLs (AMO-ZCB00, Alcon-SN60WF). Extended depth of focus (Symfony) and toric IOLs (AMO-ZCT150, AMO-ZXT150, Alcon-SN6AT) were excluded from this study.

### Assessment

Patients’ baseline characteristics (preoperative data) were recorded. Uncorrected and corrected distance visual acuity (UDVA and CDVA, respectively) were obtained 1 month after cataract surgery. All patients received a postoperative automated and manifest refraction at the end of postoperative month 1 by an ophthalmic technician who was unaware of the method used for IOL power selection, acquiring achieved spherical equivalent (SE) refractive errors. The standard 6 m (20 feet) refractive lanes were used for manifest refraction for all subjects. Predictive refractive accuracy of the preoperative and intraoperative methods was obtained by subtracting actual postoperative SE from each calculator’s predicted postoperative SE (“prediction error = achieved postoperative SE refraction—intended target SE refraction”). These prediction errors were then converted into absolute values, at which point the mean (MAE) and median (MedAE) absolute errors were obtained. Percentages of eyes within ± 0.25, ± 0.50, ± 0.75 and ± 1.00 D of intended target refraction were also calculated and compared.

### Statistical analysis

Statistical analyses were performed using Statistical Package for the Social Sciences (SPSS) version 22 (IBM, Chicago, IL, USA). Shapiro-Wilks goodness of fit test was used to determine the normal distribution of the study variables. Summary statistics were computed for continuous (mean, median) and categorical (counts, percentages) variables. T-test and ANOVA statistics for parametric analyses, Mann–whitney, Wilcoxon, MacNemar, Kruskal–Wallis statistics for non-parametric analyses were used. Chi-squared test was used to compare categorical variables. Binominal test was used to test the symmetricity of non-parametric data. Goodness of fit test was performed to check for equal distribution of categorical variables in different groups. Intra-class correlation (Cronbach’s Alpha) was used for inter-rater reliability test. ANCOVA statistics was used to control for the effect of covariate, considering required assumptions. Partial Eta squared (η^2^) was used for mean difference effect size statistics. A *P* value < 0.05 was considered statistically significant. For median comparisons, non-parametric statistical methods were used. In case of *Within-Subjects Effects* (comparing two paired samples), *Wilcoxon Signed-Rank test* was used (e.g. Tables 2, 4), and in case of *Between-Subjects Effects*, comparing two or more levels of an independent variable, *Mann–Whitney U test* and *Kruskal Wallis test* were used, respectively (e.g. Tables 3, 4). Based on our statistical analyses, we believe that the inter-eye correlation factor did not significantly affect the results of our study.

## Results

A total of 97 eyes (69 patients) with a history of myopic or hyperopic LASIK/PRK undergoing uncomplicated cataract surgery were included in this study. Of these, 81 eyes (83.5%) had previous myopic LASIK/PRK, and 16 eyes (16.5%) had previous hyperopic LASIK/PRK. The average age of patients was 67 ± 9 years old with 49.5% females and 50.5% males. Baseline characteristics of the study participants are presented in Table 1.

**Table 1:** Baseline characteristics of study participants.

	Myopic LASIK/PRK (81)	Hyperopic LASIK/PRK (16)	*P* value
Age, mean (± SD), y	66.19 (± 8.39)	73.94 (± 6.64)	*P* = 0.001*
Gender, female/male, n (%)	43(53.1%)/38(46.9%)	5(31.3%)/11(68.8%)	*P* = 0.11
Eye laterality, right/left, n (%)	45(55.6%)/36(44.4%)	11(68.8%)/5(31.3%)	*P* = 0.33
Average keratometry, mean (± SD), D	41.12 (± 2.41)	45.38 (± 1.77)	*P* < 0.001*
Axial length, median (range), mm	25.16 (22.01–30.40)	23.22 (21.61–24.74)	*P* < 0.001*
Preoperative UDVA, median, logMAR (Snellen equivalent)	0.54 (20/69)	0.40 (20/50)	0.94
Preoperative CDVA, median, logMAR (Snellen equivalent)	0.30 (20/40)	0.33 (20/43)	0.62

97 eyes were included in the study with a mean age of 67 years old. 80 eyes (82.5%) had a history of myopic LASIK/PRK and 17 eyes (17.5%) had a history of hyperopic LASIK/PRK (*P* < 0.001).

*LASIK* laser in situ keratomileusis, *PRK* photorefractive keratectomy, *UDVA* uncorrected distance visual acuity, *CDVA* corrected distance visual acuity, *logMAR* log minimum angle of resolution.

*Statistically significant.

**Figure 1:**
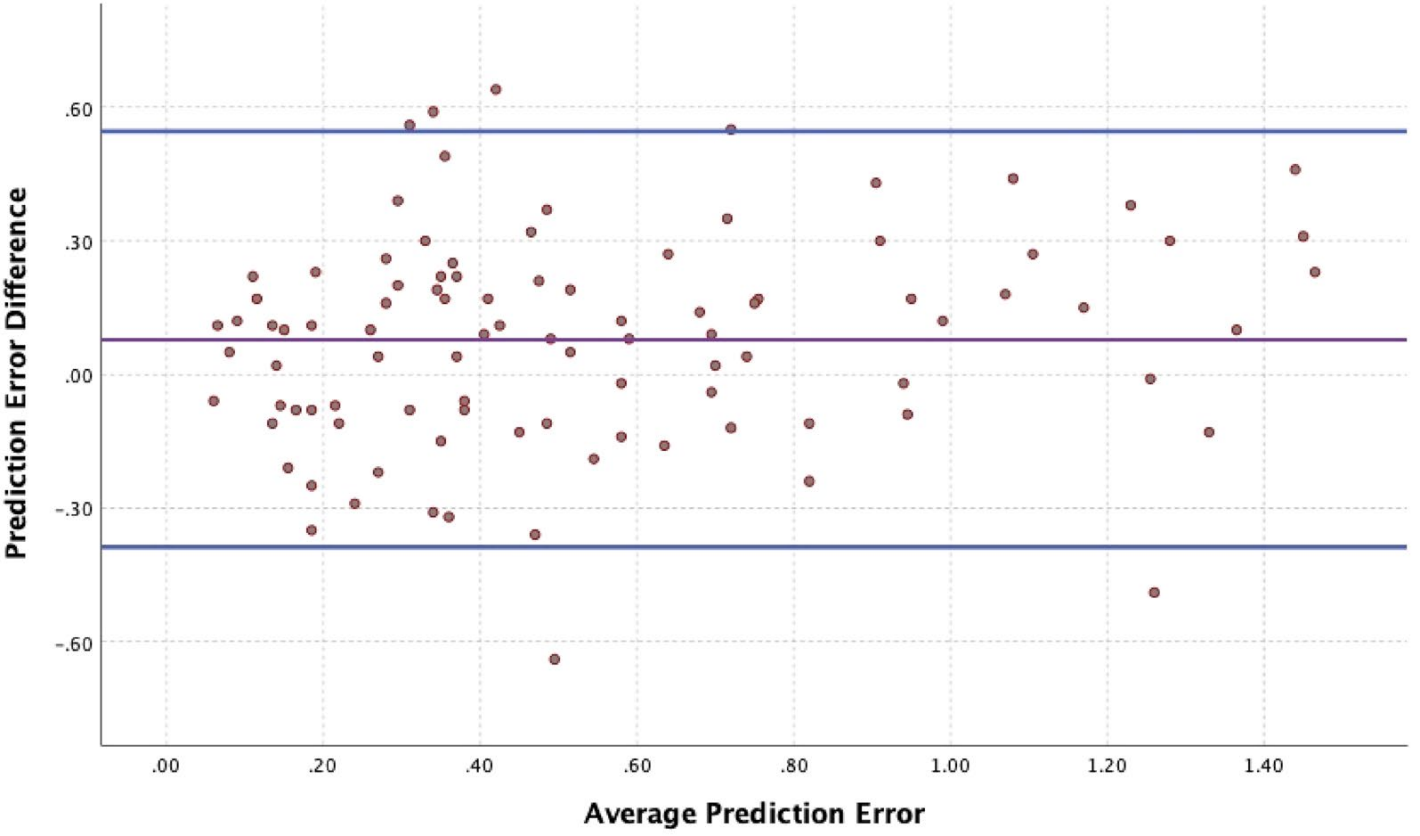
Regression analysis of Bland–Altman plot. Despite statistically significant difference between the preoperative and intraoperative methods in mean prediction error, there is good agreement between them, as demonstrated by the “Intraclass Correlation Coefficient,” Cronbach’s Alpha 0.90.

**Figure 2:**
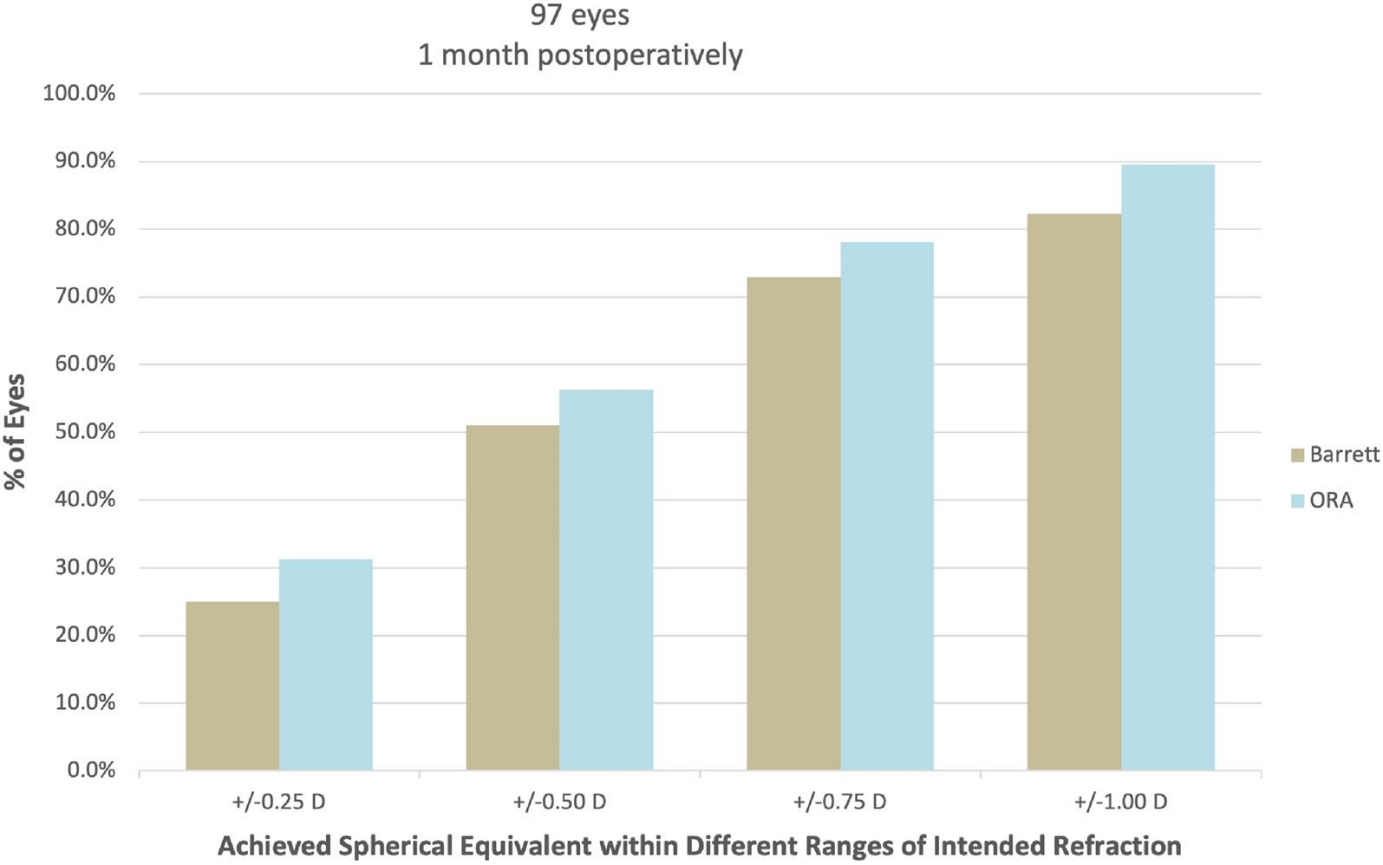
Graphical depiction of difference in achieved spherical equivalents between the preoperative and intraoperative methods. There was no statistically significant difference between the two methods for an intended target refraction up to ± 0.75 D.

Comparison between the 2 groups in predictive refractive accuracy is summarized in Table 2. As demonstrated in Table 2, the ORA system and Barrett True-K formula clinically performed comparatively in IOL power calculation. Figure 1 displays a regression analysis Bland–Altman plot showing that despite statistically significant difference between the preoperative and intraoperative methods in mean prediction error, there is good agreement between the two methods. However, the intraoperative ORA method yielded statistically significant lower median and mean absolute errors. Median and mean absolute prediction errors for the intraoperative method (ORA) were 0.42 D and 0.51 D, respectively as compared to the preoperative Barrett True-K formula, which yielded median and mean absolute prediction errors of 0.49 D and 0.58 D, respectively (*P* = 0.001/*P* = 0.002). Mean errors for Barrett and ORA methods were 0.34 D and 0.37 D, respectively. The differences between mean errors of each group and 0 were statistically significant (*P* < 0.001 for both groups). Table 2 also shows the percentage of eyes with postoperative refractions within given ranges of the target refraction for the two IOL calculation methods. There was no statistically significant difference between the two methods for an intended target refraction within ± 0.25 D, ± 0.50 D, ± 0.75 D (*P* = 0.31, *P* = 0.33, *P* = 0.18, respectively) between the two methods (Fig. 2). Percentage of eyes within ± 1.00 D of intended target refraction as predicted by the preoperative versus the intraoperative method was 82.3% and 89.6%, respectively (*P* = 0.02). ORA led to a higher number of eyes falling within ± 1.00 D of intended target refraction as compared to the Barrett True-K formula.

**Table 2:** Comparison of refractive prediction error between groups.

	PreOp method	IntraOp method	*P* value
MedAE, D (95%CI)	0.49 (0.43–0.63)	0.42 (0.33–0.54)	0.001*
MAE ± SD (D)	0.58 (± 0.41)	0.51 (± 0.37)	0.002*
Achieved SE within ± 0.25 D of intended SE, n (%)	24 (25.0%)	30 (31.3%)	0.31
Achieved SE within ± 0.50 D of intended SE, n (%)	49 (51.0%)	54 (56.3%)	0.33
Achieved SE within ± 0.75 D of intended SE, n (%)	70 (72.9%)	75 (78.1%)	0.18
Achieved SE within ± 1.00 D of intended SE, n (%)	79 (82.3%)	86 (89.6%)	0.02*

Despite a statistically significant difference between median (MedAE) and mean (MAE) absolute errors (*P* = 0.001, *P* = 0.002, respectively), the preoperative (Barrett True-K formula) and intraoperative (ORA) groups are clinically comparable in predictive refractive accuracy.

There was no statistically significant difference between the two methods for an intended target refraction up to ± 0.75 D.

Interestingly, ORA led to a higher number of eyes falling within ± 1.00 D of intended target refraction as compared to the Barrett True-K formula (*P* = 0.02).

*PreOp* preoperative method, *IntraOp* intraoperative method, *MedAE* median absolute error, *MAE* mean absolute error, *SE* spherical equivalent, *statistically significant.

**Figure 3:**
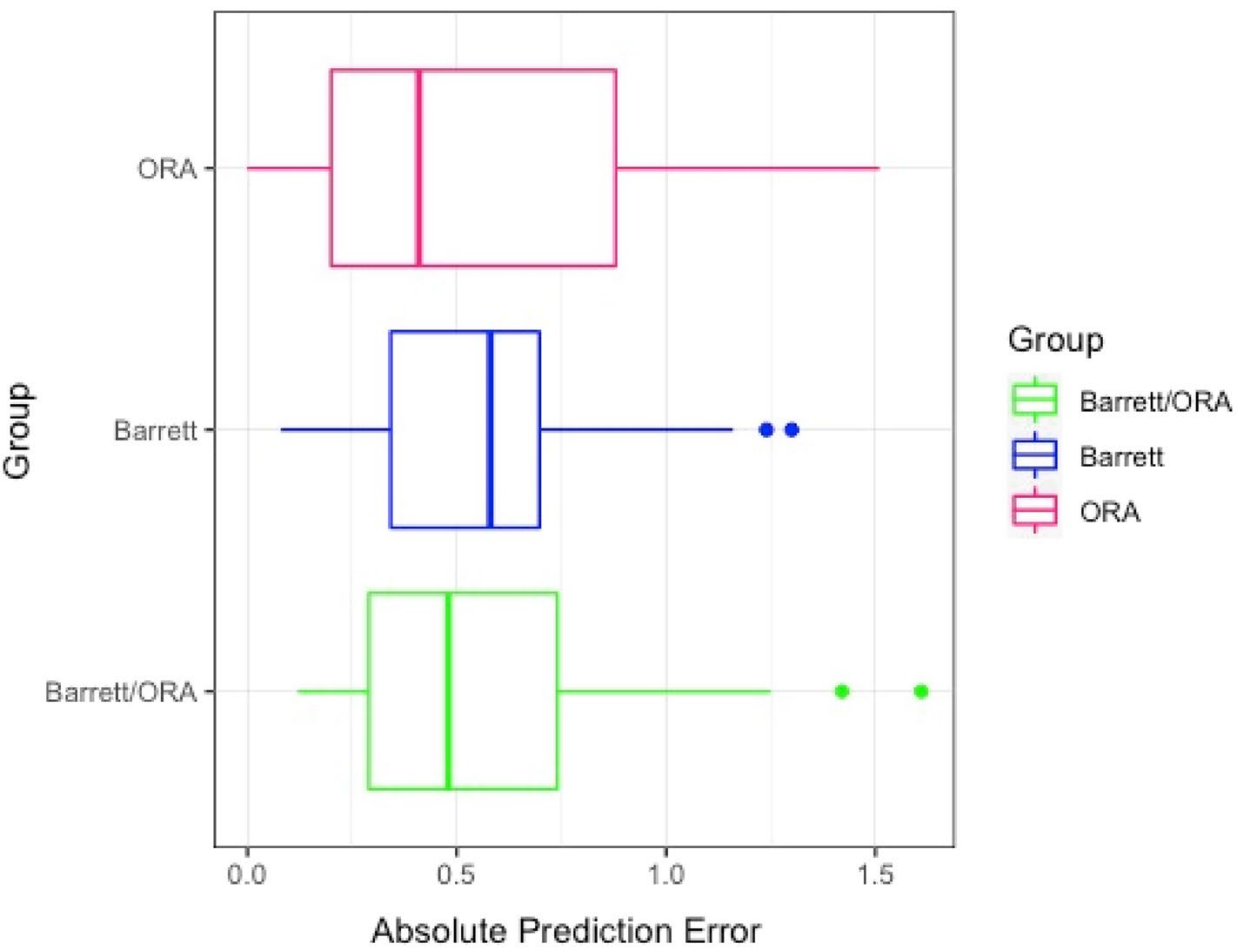
Box-plot comparison of absolute prediction error among the three groups. The median absolute error for group 3 (ORA) was lower than the other groups, although not statistically significant (*P* = 0.54). *Note*: Group 1 (Barrett/ORA): preoperative and intraoperative methods predicted same IOL power. Group 2 (Barrett): preoperative method suggestion was selected over intraoperative method. Group 3 (ORA): intraoperative method suggestion was selected over preoperative method.

In addition, eyes were grouped into one of these categories: eyes in which preoperative and intraoperative methods suggested the same IOL power where predictive refractive accuracy was calculated based on the preoperative intended target SE refraction (Group 1), eyes in which preoperative method suggestion was selected over intraoperative method suggestion (Group 2), and eyes in which intraoperative method suggestion was preferred (Group 3). Table 3 summarizes the comparison between the 3 groups in UDVA and predictive refractive accuracy parameters 1 month postoperatively. As we compared the 3 groups, while median absolute error for Group 3 was lower than other two groups, the differences were not statistically significant (*P* = 0.61) (Fig. 3). Our statistics showed only 2.2% of mean absolute error variance was explained by the selection method (preoperative vs. intraoperative) for the final IOL implantation after control for the axial length, which was not statistically significant (ANCOVA; η^2^ = 0.02; *P* = 0.58).

**Table 3:** Comparison of uncorrected distance visual acuity (UDVA) and refractive prediction error between groups.

	Group 1	Group 2	Group 3	*P* value
Number (%)	33 (34.0%)	23 (23.7%)	41 (42.3%)	0.08
Postoperative UDVA, median, logMAR (Snellen equivalent)	0.097 (20/25)	0.176 (20/30)	0.176 (20/30)	0.28
MedAE (95%CI), D	0.48 (0.34–0.68)	0.58 (0.31–0.71)	0.41 (0.26–0.67)	0.61
MAE (± SD), D	0.56 (± 0.37)	0.59 (± 0.36)	0.54 (± 0.42)	0.87
Achieved SE within ± 0.25 D of intended SE, n (%)	6 (18.2%)	4 (18.2%)	13 (31.7%)	0.31
Achieved SE within ± 0.50 D of intended SE, n (%)	18 (54.5%)	10 (45.5%)	24 (58.5%)	0.61
Achieved SE within ± 0.75 D of intended SE, n (%)	26 (78.8%)	17 (77.3%)	28 (68.3%)	0.55
Achieved SE within ± 1.00 D of intended SE, n (%)	28 (84.8%)	18 (81.8%)	34 (82.9%)	0.95

There was no statistically significant difference in refractive prediction error 1 month post-operatively among the groups.

*Group 1* preoperative same as Intraoperative intraocular lens power suggestion, *Group 2* preoperative suggestion selected, *Group 3* intraoperative suggestion selected, *UDVA* uncorrected distance visual acuity, *logMAR* log minimum angle of resolution, *MedAE* median absolute error, *CI* confidence interval, *MAE* mean absolute error, *SE* spherical equivalent.

Furthermore, stratified analysis of the performance of the 2 methods based on type of refractive surgery is summarized in Table 4. We found that IOL power measurement was better predicted in myopic LASIK/PRK by the intraoperative method as demonstrated by the mean absolute error (*P* < 0.01). However, we did not find any interaction between IOL power measurement method and hyperopic LASIK/PRK surgery in mean absolute prediction error (*P* = 0.10). Our study showed that the preoperative method performed better among hyperopic refractive surgery patients than myopic patients in terms of median and mean absolute error (Post Hoc for mean comparison; *P* = 0.04) with statistically strong effect size (η^2^ = 0.14).

**Table 4:** Comparison of predictive refraction errors based on refractive surgery type.

	Myopic LASIK/PRK (81)			Hyperopic LASIK/PRK (16)			Between groups (refractive surgery Types) *P* values	
	PreOp	IntraOp	*P*	PreOp	IntraOp	*P*	PreOp	IntraOp
MedAE	0.54	0.47	< 0.01*	0.35	0.24	0.22	0.09	0.02*
MAE	0.62	0.54	< 0.01*	0.41	0.31	0.19	< 0.01*	0.02*

The intraoperative method performed better than the preoperative method in predictive refractive accuracy in patients with a history of myopic LASIK/PRK in comparison to those with a history of hyperopic LASIK/PRK.

*LASIK* laser in situ keratomileusis, *PRK* photorefractive keratectomy, *MedAE* median absolute error, *MAE* mean absolute error, *PreOp* preoperative method, *IntraOp* intraoperative method; *statistically significant.

## Discussion

To our knowledge, this is one of the few studies that compares the preoperative APACRS Barrett True-K formula to the IA (the ORA System) in predictive refractive accuracy of IOL power calculation in patients with prior refractive surgery. In our study, although the ORA system achieved a lower median and mean absolute error and achieved a spherical equivalent closer to the intended target refraction, both the ORA system and the Barrett True-K formula clinically performed comparatively in predictive refractive outcomes. Both of these methods can be effective in selection of IOL power in patients with prior myopic or hyperopic LASIK or PRK without historical corneal data.

Several strategies have been proposed to overcome the challenge in IOL power selection in an effort to improve refractive outcomes in post-LASIK/PRK patients undergoing cataract surgery. Historical data pertaining to pre-refractive surgery data is often limited as the data may be difficult to obtain. Other options include methods that calculate corneal power to determine IOL power selection. Standard keratometers and placido disk-based corneal topography devices measure the corneal curvature several millimeters away from the small, effective central optical zone resulting from previous refractive surgery*.* Hence, postoperative hyperopic surprise used to be more common especially in patients with previous myopic refractive surgery due to overestimation of corneal power^9^. Most standard corneal measurement devices do not take the posterior cornea into account, and thus calculate the anterior corneal curvature instead. As the relationship between the anterior and posterior corneal curvatures may be considerably altered following LASIK or PRK*,* such changes may lead to poor visual outcomes^15^. The Barrett True-K formula is one of the more recently developed methods and can be used without historical data. Studies comparing it to other formulas in patients with previous LASIK or PRK suggest it was at least equal to and often better than other methods with accurate refractive results^11,21–24,28^. However, studies comparing the Barrett True-K formula to IA may reveal conflicting results.

Using newer technologies such as the ORA System has made real-time intraoperative refraction and IOL power calculation feasible. Compared to limitations reported in conventional methods of IA such as the Hartmann-Shack method with limited dynamic range and variations of the readings^16^, newer technology of Talbot-Moiré interferometry has been associated with more reliable results. This is especially important for more challenging cases such as patients with history of refractive surgery. Undoubtedly, IA does have its own limitations. Potential factors during surgery may affect the precision of measurements e.g. eyelid squeezing or extraocular movements by the patient during surgery, pressure and/or distortion induced by an eyelid speculum, the effect of certain ophthalmic viscosurgical devices and corneal stromal hydration intraoperatively^16,17^.

In our study, intraoperative ORA method yielded statistically significant lower MedAE and MAE than the preoperative Barrett True-K formula. The MedAE and MAE for the intraoperative method (ORA) was 0.42 and 0.51, respectively as compared to the preoperative Barrett True-K formula, which yielded MedAE and MAE of 0.49 and 0.58, respectively (*P* = 0.001/*P* = 0.002). The percentage of eyes within ± 1.00 D of intended target refraction as predicted by the ORA system versus the Barrett True-K formula was high, 89.6% and 82.3%, respectively (*P* = 0.02). ORA led to a higher number of eyes falling within ± 1.00 D of intended target refraction as compared to the Barrett True-K formula, although there seemed to be no statistically significant difference between the two methods for an intended target refraction within ± 0.25 D, ± 0.50 D, ± 0.75 D (*P* = 0.31, *P* = 0.33, *P* = 0.18, respectively). In concordance with our study, a retrospective case series of 173 eyes by McCarthy *et al*. reported that by using different published methods of IOL power calculation after myopic laser refractive surgery, 70% to 85% of eyes could achieve visual outcomes within ± 1.00 D of target refraction^10^. In another prospective study of 104 eyes, Wang *et al*. reported promising results using newer formulas of IOL power calculation, such as optical coherence tomography (OCT)-based and the Barrett True-K formula in patients with previous myopic LASIK or PRK^11^. Similarly, Ianchulev *et al*. demonstrated that IA can be successfully used in patients with prior refractive surgery especially when historical data is not available^8^. In their retrospective study of 246 eyes with prior myopic LASIK or PRK, they showed that the ORA system achieved the greatest predictive accuracy compared to preoperative methods (the surgeon’s choice based on available clinical data, the Haigis L, and the Shammas IOL formulas). They reported median absolute error of 0.35 D for the intraoperative method with 67% of eyes within ± 0.50 D of prediction error. In another study with eyes that had undergone previous laser vision correction (LVC), Fram *et al*. reported promising results using newer technologies (such as ORA intraoperative aberrometry or Optovue RTVue Fourier-domain OCT-based IOL formula) to estimate IOL power when compared with established methods^12^. In their study, 69–74% of eyes were within ± 0.50 D of intended target refraction among patients without historical data. Contrarily, another study by Dawson *et al*. noted no statistically significant difference between Barrett True-K and IA in predicting postoperative refractive error although their study included eyes with prior RK^26^. A study by Christopher *et al*. also showed no difference between Barrett True-K and IA in predicting postoperative refractive error within ± 0.50 D of intended target in patients with myopic refractive surgery although they observed statistical improvement in prediction outcome from 69 to 72% when posterior cornea measurements were utilized in Barrett True-K formula^29^.

There have also been reports of IA calculations in patients without a history of refractive surgery. In a recent report of a large retrospective analysis on 32,189 eyes without prior refractive surgery, Cionni *et al*. reported IA calculations outperformed preoperative calculations; they also emphasized that the difference was more pronounced in cases in which the preoperatively planned IOL power was different than the power of the IOL implanted^13^. Zhang *et al*. reported comparable results of refractive prediction for ORA and IOL master measurements in patients without prior refractive surgery^14^. They also stated that the highest predictive accuracy is in cases that ORA and IOL master recommended the same IOL power. Although our data showed lower median/mean absolute error for Group 3, as seen in Table 3 and Figure 3, there are overlapping confidence intervals, and the differences were not statistically significant. It may be that the study by Zhang *et al.* excluded all patients with prior refractive surgery, so the conclusions from their study may not apply to our study population. A more recent study of 949 eyes by Raufi *et al*. found that refractive outcomes for patients without a history of refractive surgery was not improved by utilizing IA^25^. In further agreement was another study by Sandoval *et al*., which found that IA results were not significantly different than the Barrett True-K formula within 0.50 D or 1.00 D (*P* > 0.2)^27^. In our study of patients with a history of refractive surgery, results revealed that using the ORA System suggestion for IOL power selection was associated with slightly lower MedAE and MAE compared to the Barrett True-K formula (*P* = 0.001), although the two methods are clinically comparable.

We also found that IOL power measurement was better predicted in myopic LASIK/PRK by the intraoperative method as demonstrated by the MedAE and MAE (*P* = 0.001). However, we did not find any interaction between IOL power measurement method and hyperopic LASIK/PRK. Our study showed that both methods performed better among hyperopic refractive surgery patients than myopic patients in terms of median and mean absolute error. In a retrospective study of 46 eyes, Canto *et al*. showed that by using Orange (the older generation of IA; WaveTec Vision Systems Inc.), 37% of eyes reached ± 0.50 D of emmetropia compared to 30% for IOL Master^18^. In a study by Chean *et al*., the differences between the intended and postoperative refractive error was greater in post-LVC eyes than the control eyes, irrespective of which method was used to calculate the intended refractive error (*P* < 0.01)^19^. A study by Gouvea *et al.* which included patients with RK, hyperopic/myopic laser vision correction, refractive lens exchange, toric/EDOF/multifocal IOLs in their analysis concluded intraoperative aberrometry performed better in patients with prior hyperopic LVC in contrary to our conclusions^30^. Further investigation regarding the the role of these new methods, especially in patients with previous myopic or hyperopic LASIK/PRK is warranted in future studies.

The current study has some limitations. First, a small sample size limits the generalizability and power of our study. Power analysis was not performed prior to data collection because all available data from our institution were utilized and post hoc power analyses have not been found to be useful. Second, our study had more patients with a history of myopic versus hyperopic LASIK/PRK and did not include patients with a history of RK. Based on Mixed ANOVA analysis, we did not find any interaction between IOL power measurement method (Barrett vs. ORA) and refractive surgery type (myopic vs. hyperopic) in mean absolute prediction error (*P* = 0.37) with our current dataset and decided to present our data in aggregate for Tables 2 and 3. In future studies, this lack of interaction may not hold as additional post hyperopic LVC patients are added to the analysis. Furthermore, IA in aphakic eyes is more reliable once stable and pressurized anterior chamber conditions are achieved^20^. Throughout intraoperative refraction, we tried to establish normal intraocular pressure using a cohesive viscoelastic agent, and check ocular pressure before measurements. Some errors might still be encountered due to variability of these factors such as intraocular pressure, corneal hydration, and/or external ocular pressure at the time of intraoperative refraction. Our results were also based on multiple cataract surgeons, and although there may be variability in application of the results, given that cataract surgery has become streamlined, this may be a minor limitation; contrarily, this makes the applicability of our study results to other surgeons more feasible. Another limitation includes length of follow-up for our patients since postoperative refraction for our study was obtained at the 1-month visit. Some surgeons feel we should wait at least 3 months after surgery to evaluate the final manifest refraction for post-refractive surgery patients due to continued corneal changes. Lastly, postoperative refraction may be subjective and dependent on both the patient and the examiner, and although increasingly precise, our calculations were not based on the results of wavefront refraction. Prospective studies with standardization of these factors are warranted to obtain more conclusive results.

## Conclusion

In conclusion, our study results indicate that the use of IA may have a small advantage in IOL power calculation in eyes with a history of corneal refractive surgery. Overall, IA (the ORA System) and the Barrett True-K formula are clinically comparable in IOL power calculation in patients with prior refractive surgery. Additional studies may be beneficial to establish their role amongst these challenging patients.

## Data Availability

The datasets analyzed for this study are available from corresponding author on reasonable request.
